# Suppression of neuropathic pain and comorbidities by recurrent cycles of repetitive transcranial direct current motor cortex stimulation in mice

**DOI:** 10.1038/s41598-021-89122-6

**Published:** 2021-05-06

**Authors:** Zheng Gan, Han Li, Paul Vincent Naser, Manfred Josef Oswald, Rohini Kuner

**Affiliations:** Institute of Pharmacology, Medical Faculty Heidelberg, Heidelberg University, Im Neuenheimer Feld 366, 69120 Heidelberg, Germany

**Keywords:** Neuroscience, Somatosensory system, Pain, Chronic pain

## Abstract

Transcranial, minimally-invasive stimulation of the primary motor cortex (M1) has recently emerged to show promise in treating clinically refractory neuropathic pain. However, there is a major need for improving efficacy, reducing variability and understanding mechanisms. Rodent models hold promise in helping to overcome these obstacles. However, there still remains a major divide between clinical and preclinical studies with respect to stimulation programs, analysis of pain as a multidimensional sensory-affective-motivational state and lack of focus on chronic phases of established pain. Here, we employed direct transcranial M1 stimulation (M1 tDCS) either as a single 5-day block or recurring blocks of repetitive stimulation over early or chronic phases of peripherally-induced neuropathic pain in mice. We report that repeated blocks of stimulation reverse established neuropathic mechanical allodynia more strongly than a single 5-day regime and also suppress cold allodynia, aversive behavior and anxiety without adversely affecting motor function over a long period. Activity mapping revealed highly selective alterations in the posterior insula, periaqueductal gray subdivisions and superficial spinal laminae in reversal of mechanical allodynia. Our preclinical data reveal multimodal analgesia and improvement in quality of life by multiple blocks of M1 tDCS and uncover underlying brain networks, thus helping promote clinical translation.

## Introduction

A large number of clinical cases of neuropathic pain remain refractory to pharmacological treatment or their use is hampered by serious side effects at dosage regimens required for adequate pain relief^1,2^. This has led to an accruing interest in non-pharmacological alternatives, amongst which neurostimulation-based therapies are most prominent^3–6^.

Use of neurostimulation and neuromodulation was pioneered in the motor cortex, and a significant body of literature has emerged from both experimental and therapeutic paradigms for stimulation of the primary motor cortex (M1)^5,7–9^. Although initial focus was placed on invasive deep brain M1 stimulation, transcranial direct current stimulation (tDCS) and magnetic stimulation (TMS) rapidly took upper hand owing to their less invasive nature, safety, ease of administration and lower cost^10–12^. Detailed evidence-based guidelines for use of M1 stimulation in therapeutic management of chronic pain disorders were developed by expert panels in 2014^6^ and very recently up-dated in 2020^13^, which indicate a definite efficacy for contralateral M1 stimulation for neuropathic pain and probable efficacy for left M1 stimulation for improving quality of life in fibromyalgia patients.

While there is substantial evidence for efficacy in management of certain types of pain disorders with M1 tDCS and TMS, there is also considerable variability; particularly, their use in everyday clinical practice is still limited owing to scarcity of standardized treatment regimens, potential of side effects as well as lack of knowledge on underlying mechanisms and their long-term ramifications^3,6,10–12,14^. Rodent models can make valuable contributions in addressing mechanisms and brain pathways underlying analgesia induced by motor cortex stimulation. Indeed, a number of high-quality studies have successfully modelled modulation of nociception by MCS in acute and chronic pain models and have been particularly insightful in addressing how basal (resting) activity of key pain-related centers changes in the brain and brain-spinal cord axis^9,15–17^.

A number of critical gaps remain in modelling MCS from diverse perspectives in animal models of chronic pain and in our understanding of underlying mechanisms, which are decisive for promoting translational relevance and applicability to the clinical counterparts^3,4^. For example, while clinical studies point to the need of a large number of repetitions of MCS for achieving efficacy in treating pain, most animal studies employ simple regimens which employ single stimulation session over a short time window, do not reapply repetitions of stimulation cycles, and target either basal nociception or a particular phase of a chronic pain disorder^15,16,18^. Secondly, most animal studies target a single phase of a pain disorder, which is largely acute or sub-acute^19–21^; in contrast, patients undergoing MCS typically fall in the category of late stage, chronically established neuropathic pain, rendering translation of insights difficult. Importantly, while neuropathic pain in patients is a complex mixture of diverse sensory abnormalities over several modalities, changes in pain affect and comorbidities, such as anxiety and fear, most animal studies so far have primarily tested the impact of MCS on sensory qualities of pain, i.e. nociception^15,16,18,19,22,23^. Overall, this remains a nascent, emerging field in comparison to the vast number of studies and massive volume of data available on pharmacological interventions in chronic pain^2,24^.

In the light of these open questions, the current study was designed to test the functional impact of testing repeated blocks of anodal M1 stimulation over distinct phases of neuropathic pain, including early stages and late stages when neuropathic pain is firmly established over a chronic period. Secondly, we addressed efficacy in diverse sensory and affective parameters of chronic pain and studied potential side effects on motor function. While a few previous studies have provided important insights on brain areas modulated by MCS in single use paradigms^15,18,23,25^, we addressed how the activity of pain-related brain areas changes over repeated use of MCS cycles. Finally, while changes in resting brain activity has been studied previously^16,26–28^, this study also addresses a hitherto unstudied question, namely the identity of regions and nuclei in the brain, brainstem and spinal cord that specifically underlie MCS-induced reversal of neuropathic mechanical allodynia, i.e. circuits recruited by innocuous touch that is perceived as painful in neuropathic conditions. Thus, the study delivers promising insights on clinically-relevant issues that may help improve the translation of preclinical knowledge to improving MCS-based therapies.

## Results

### Inhibition of progression of mechanical hypersensitivity in early stage neuropathic pain by anodal M1 tDCS

**Figure 1:**
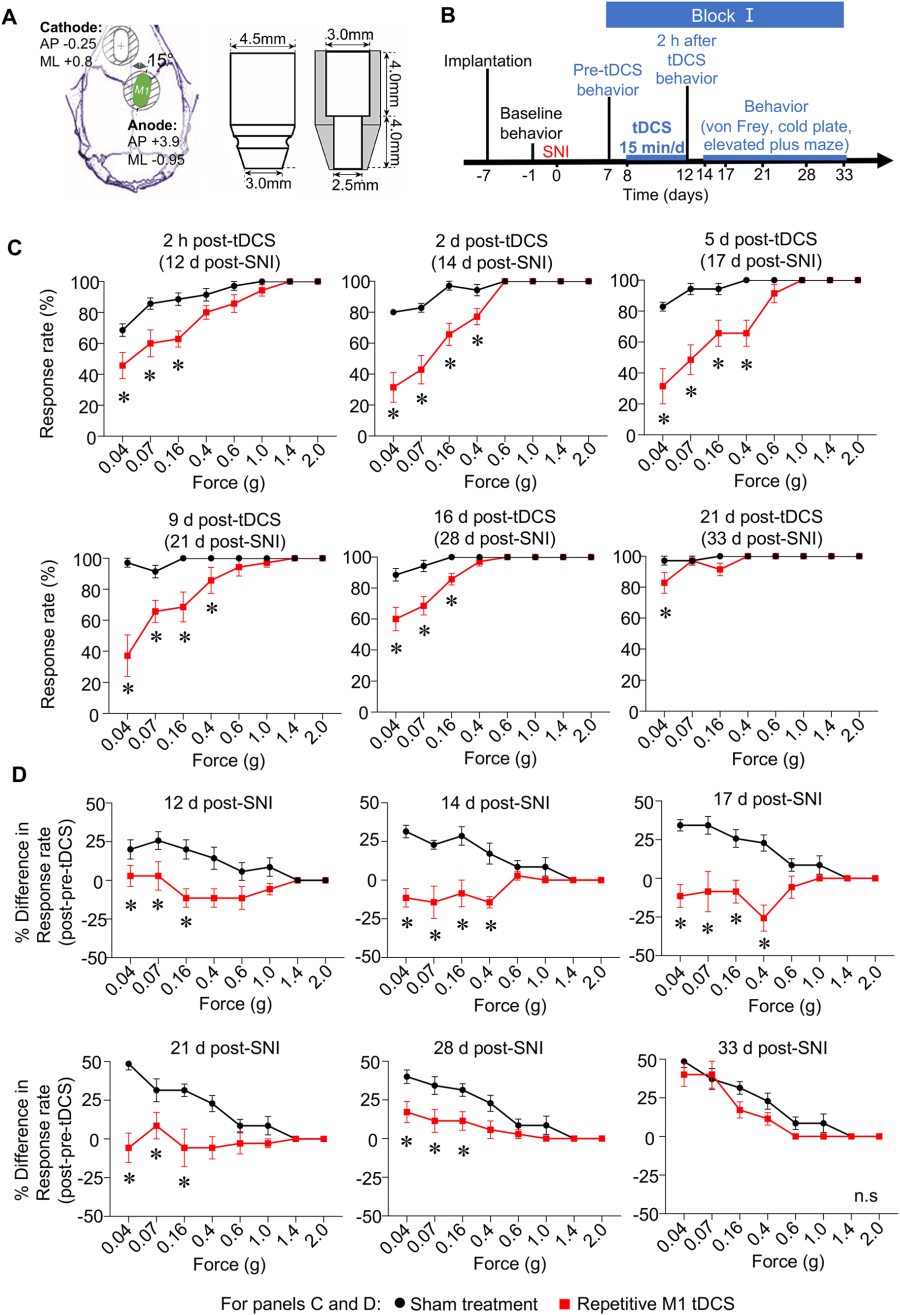
Suppression of progression of mechanical allodynia in neuropathic mice upon 5 cycles of anodal transcranial direct current stimulation (tDCS; Block I) of the primary motor cortex (M1) applied over early stages post-nerve injury (SNI). (**A**) Schematic illustration of placement of transcranial electrodes over the skull with anterio-posterior (AP) and medio-lateral (ML) coordinates relative to Bregma and electrode dimensions. (**B**) Schematic overview on the experimental plan with electrode implantation, nerve injury, stimulation protocol and behavioral analyses at baseline (pre-SNI), pre-tDCS and longitudinally until 33 days post-SNI. (**C,D**) Behavioral analysis of withdrawal response rate to 5 applications of graded force via plantar mechanical von Frey stimulation (**C**). Average difference in response rate following tDCS or sham treatment as compared to pre-stimulation values within each animal are shown in (**D**). In panels (**C,D)**, n = 7 mice/group; ANOVA for repeated measures was performed, followed by Sidak’s test for multiple comparisons; *indicates *p* < 0.05 as compared to the corresponding control group. Data are represented as mean + /− standard error of the mean (S.E.M).

In the first set of behavioral experiments, we behaviorally analyzed the impact of tDCS via anodal electrodes placed over the murine primary motor cortex (M1; Fig. 1A) contralateral to nerve injury over early stages as per the experimental scheme outlined in Fig. 1B. Implanted mice were subjected to baseline assessment of nociceptive sensitivity to mechanical and cold stimuli applied to the paw, subjected to nerve injury in the Spared Nerve Injury (SNI) model of neuropathic pain^29^ and the neuropathy-induced decrease in response rate and threshold to mechanical stimuli and cold temperatures was recorded in the sural territory of the hind paw (pre-tDCS behavior in Fig. 1B). In the first set of mice (Block I), anodal M1 tDCS was performed at 0.35 mA current (repetitive M1 tDCS group) or 0 mA current (sham treatment group) at 5 daily repetitions for 15 min over a period of 5 days. We recorded nociceptive sensitivity starting at 2 h after the last bout of stimulation and until 3 weeks thereafter. Baseline sensitivity before SNI (Suppl. Figure 1B), before tDCS at 6 days post-SNI (Suppl. Figure 1C) was similar across both groups. Neuropathic mice in both sham- and tDCS- groups showed a significant enhancement in the response rate, particularly to typically non-noxious intensities of mechanical von Frey stimulation (0.04–0.16 g; mechanical allodynia) as compared to pre-SNI behavior (Suppl. Figure 1B, Fig. 1C). However, already starting at 2 h after the last session of stimulation, tDCS mice demonstrated significantly lesser mechanical allodynia than sham-stimulated mice (Fig. 1C). This first block of repetitive tDCS provided significant relief from mechanical allodynia for up to 16 days post-stimulation (Fig. 1C). Importantly, over the early phase after nerve injury (12 days post-SNI), mice from the sham stimulation group continued to develop more mechanical hypersensitivity when tested until 33 days post-SNI, as seen upon plotting the difference in response rate between post- and pre-stimulation in the sham group (Fig. 1D). In contrast, mechanical hypersensitivity in mice from the tDCS group did not exacerbate over 12–28 days post-SNI and the beneficial effect only wore off at 33 days post-SNI (21 days post-tDCS). These results demonstrate that anodal M1 tDCS not only lowers the magnitude of mechanical hypersensitivity, but also significantly protects against the progression of neuropathic allodynia over early stages post-nerve injury.

### Effects of reapplication of M1 tDCS at chronic stages of neuropathic mechanical allodynia

**Figure 2:**
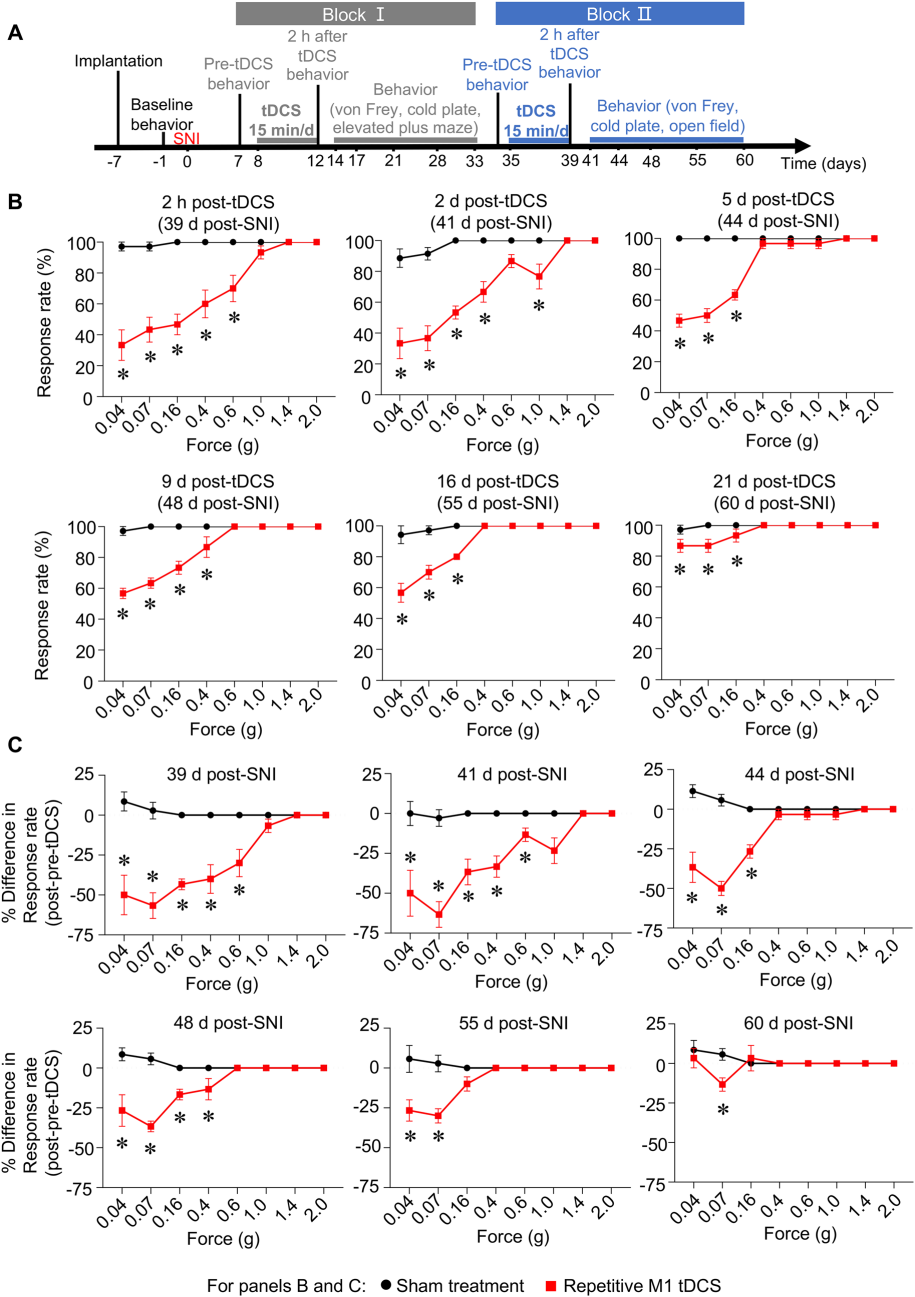
Reversal of established, peak mechanical allodynia in chronic neuropathic mice upon a second application of 5 cycles of anodal M1 tDCS (Block II) in mice over late stages post-nerve injury (SNI). (**A**) Schematic overview on the longitudinal behavioral analyses starting from 41 to 60 days post-SNI following Block II M1 tDCS over 35–39 days post-SNI. (**B**,**C**) Behavioral analysis of withdrawal response rate to 5 applications of graded force via plantar mechanical von Frey stimulation (**B**). Average difference in response rate following tDCS or sham treatment as compared to pre-stimulation values within each animal are shown in (**C**). In panels (**B,C)**, n = 6 mice for the sham treatment group and n = 7 mice for repetitive M1 tDCS group; ANOVA for repeated measures was performed, followed by Sidak’s test for multiple comparisons; *indicates *p* < 0.05 as compared to the corresponding control group. Data are represented as mean + /− standard error of the mean (S.E.M).

Once the protective effects of the first block of repetitive tDCS had worn off and mechanical hypersensitivity was fully restored in the chronic stage, we then tested the potential of a second block of stimulation in alleviating different sensory and emotional/affective aspects of chronic neuropathic pain (Block II, Fig. 2A). First, we observed that the second block of stimulation exerted an even greater decrease in mechanical allodynia starting from 2 h after the last cycle of stimulation (i.e. 39 days post-SNI; Fig. 2B) and lasting until 21 days post-tDCS (i.e. 60 days post-SNI; Fig. 2B) than the protection observed in Block I (Fig. 1C). A marked reversal of maximally established mechanical hypersensitivity was observed with the second block of tDCS when difference in response rate between post- and pre-stimulation was analyzed over all time points (Fig. 2C). This indicates that tDCS-induced protection is potentiated upon repeating blocks of 5-day tDCS cycles over the course of neuropathic pain.

### Comparison of impact of single and recurrent blocks of repetitive tDCS cycles on neuropathic cold allodynia

**Figure 3:**
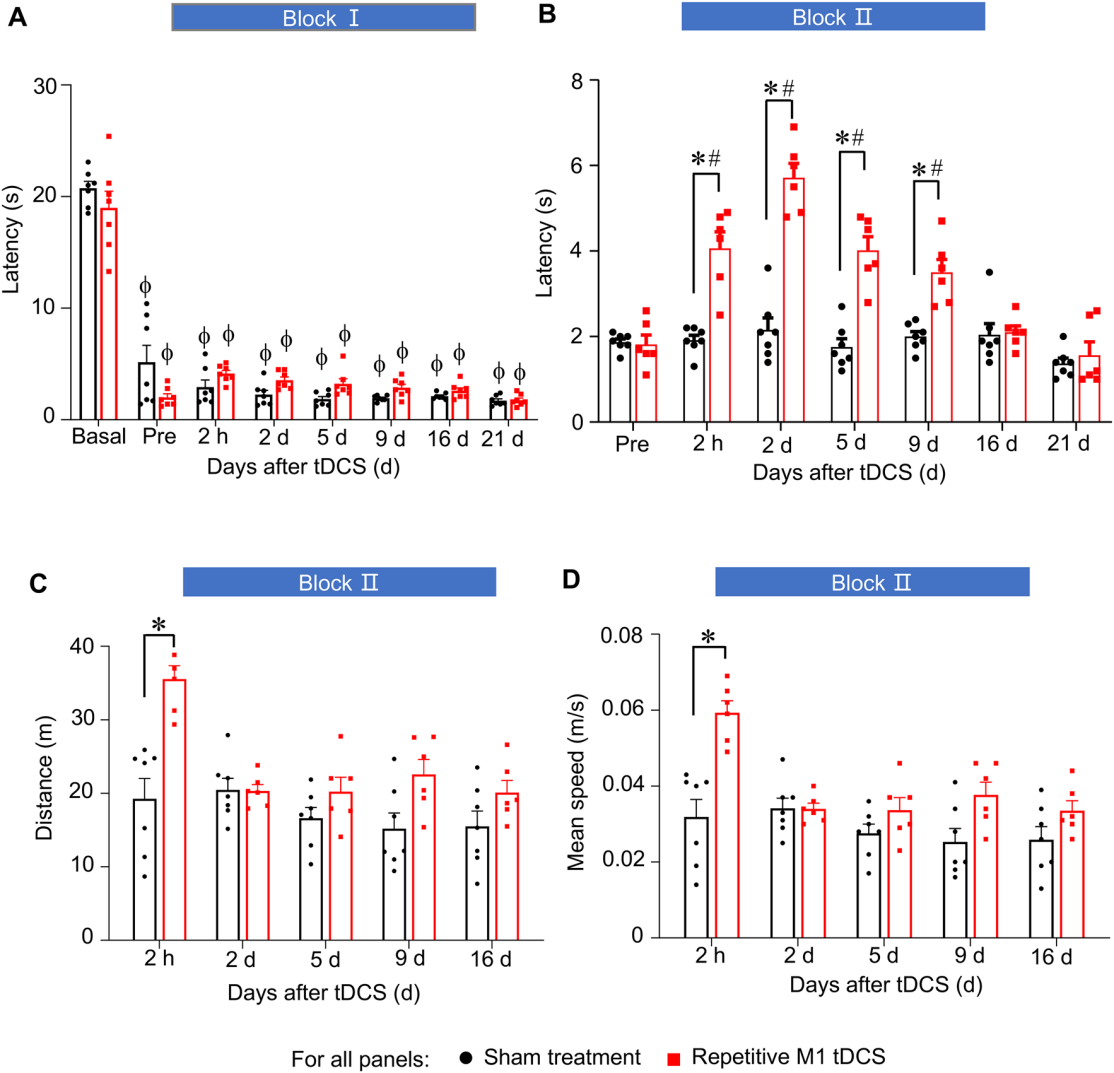
Comparison of the impact of the application of Block I and Block II anodal M1 tDCS over early and the chronic phase of neuropathic pain post-nerve injury (SNI), respectively, on cold allodynia and motor function. (**A,B**) Partial, significant reversal of allodynia to cold, shown in form of latency of nociceptive responses to 5° temperature, by repetition of anodal M1 tDCS in chronic phase post-SNI (Block II), but not by the first 5-day cycle of M1 tDCS over the early phase post-SNI (Block I) in comparison with the respective sham treatment group on. (**C,D**) Early and short-lasting enhancement of locomotion parameters by anodal M1 tDCS (Block II). n = 7 mice for the sham treatment group and n = 6 mice for repetitive M1 tDCS group; ANOVA for repeated measures was performed, followed by Sidak’s test for multiple comparisons; *indicates *p* < 0.05 as compared to the corresponding sham group, # indicates *p* < 0.05 as compared to the pre-stimulation value within each group. ϕ indicates *p* < 0.05 as compared to the basal value within each group Data are represented as mean + /− S.E.M.

A hallmark clinical feature of neuropathic pain is given by marked hypersensitivity to cold stimuli, which is particularly debilitating in patients with nerve injury in cooler climates^2,30^. This important characteristic has been seldom studied in rodent analyses on transcranial motor cortex stimulation. Mice with SNI demonstrated a sharp fall in latency of withdrawl response to a mild temperature of 5 °C on a cold plate as compared to pre-SNI values (Fig. 3A). The first block of M1 tDCS had no impact on cold allodynia when behavior was compared between the tDCS and sham groups until 21 days post-stimulation (Fig. 3A). In contrast, the second block of the 5-day tDCS cycle elicited a moderate, but significant, decrease in cold allodynia starting from 2 h after the last stimulation and lasting up to 9 days (Fig. 3B).

Importantly, neither the suppression of withdrawal behaviors related to mechanical nor cold allodynia resulted from impairment of motor impairment. This was evident when we performed control experiments on mice after the second block of repetitive M1 tDCS testing locomotion parameters, such as distance travelled (Fig. 3) and mean speed (Fig. 3), in an open field. SNI mice with M1 tDCS did show an enhancement of motor function immediately following the last bout of stimulation in the 5-day cycle (as compared to mice with SNI mice with sham stimulation Fig. 3C,D). However, this beneficial effect did not last long, with no differences being detected between groups between 2 and 16 d post-stimulation.

### Recurrent blocks of repetitive tDCS suppress neuropathic pain-related operant behaviors and anxiety

**Figure 4:**
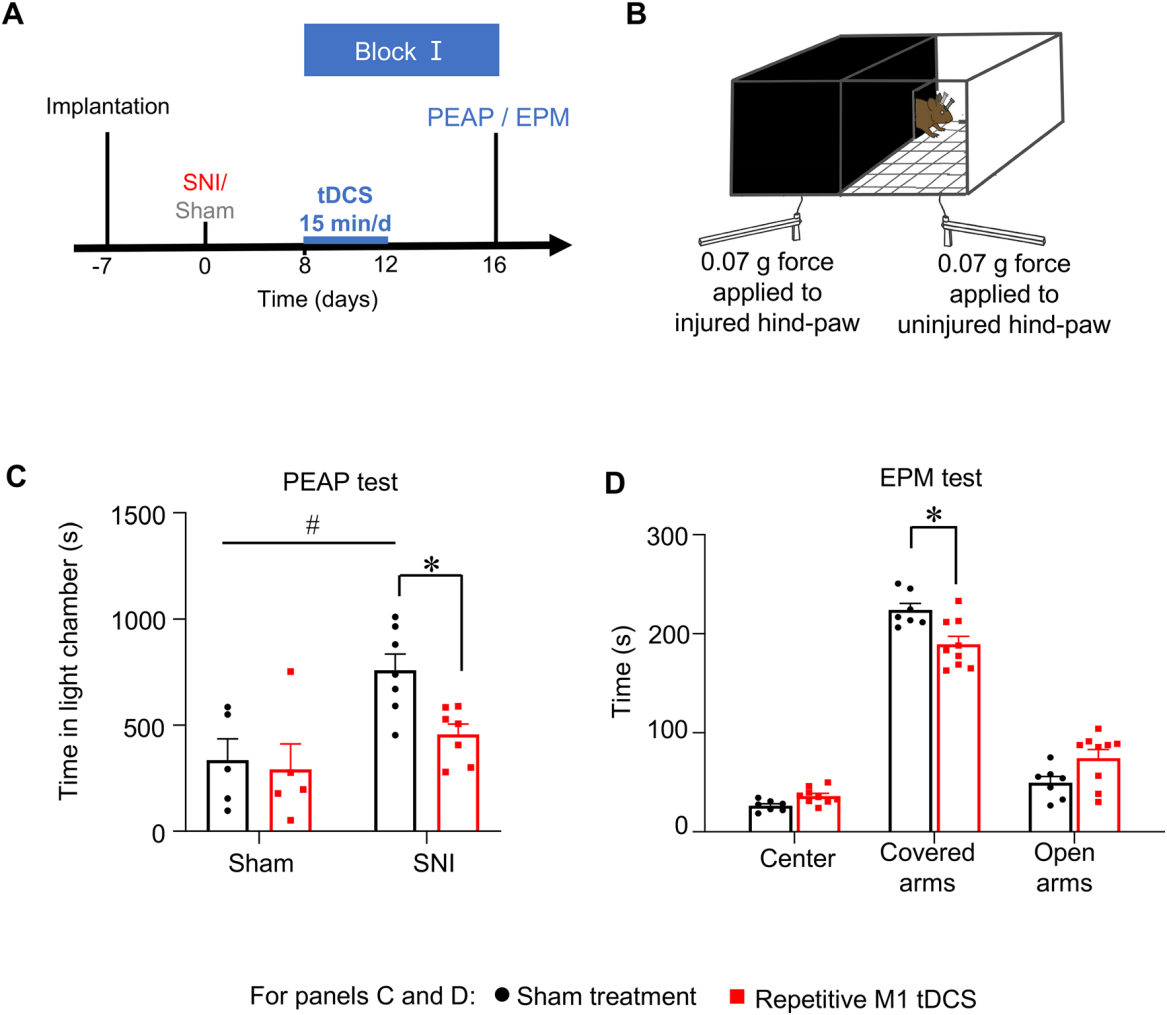
Impact of anodal M1 tDCS on behavioral parameters of neuropathic pain-associated aversion and anxiety. (**A**) Experimental scheme of implantation, nerve injury, stimulation protocol and behavioral analyses. (**B**) Scheme of the place escape avoidance paradigm (PEAP test), whereby light tactile von Frey force (0.07 g) is applied to the injured hind paw in the dark side of the chamber, while the uninjured hind paw of the mouse was given the same stimulus in the light side of the chamber in neuropathic mice; n = 5 mice each for the sham surgery groups and n = 7 mice each for the SNI groups (**C**) Quantitative summary of time spent in the light chamber (i.e. avoiding the dark chamber) in SNI and sham surgery groups of mice with M1 tDCS and sham stimulation treatment. (**D**) Analysis of anxiety, demonstrated as time spent in covered arms, in the elevated plus maze test in neuropathic mice with M1 tDCS and sham control treatment; n = 7 mice for the sham treatment group and n = 9 mice for repetitive M1 tDCS group. In all experiments, ANOVA for random measures was performed, followed by Tukey’s test for multiple comparisons, * indicates *p* < 0.05 as compared to the corresponding sham group. Data are represented as mean ± S.E.M.

The place escape avoidance behavior (PEAP) test enables studying active avoidance of harmful or aversive stimuli in awake, freely-moving mice independently of involuntary reflexive behaviors (longitudinal scheme in Fig. 4A, schematic representation of PEAP test in Fig. 4B). When we applied a mechanical von Frey stimulus of 0.07 g, corresponding to light touch, in a dark chamber to the paw ipsilateral to nerve injury, SNI mice with sham stimulation avoided the dark chamber and spent more time in a bright chamber which they would have typically shunned (Fig. 4B). This behavior is indicative of mechanical allodynia and aversion to light touch^31^. In comparison with sham-stimulated mice, SNI mice with tDCS treatment spent considerably less time in the bright chamber, i.e. avoiding the dark chamber associated with aversive tactile mechanical stimulation (Fig. 4C). These results demonstrate that the affective, aversive component of neuropathic pain is also significantly suppressed by repetitive M1 tDCS.

To address anxiety related to neuropathic pain, we employed the elevated plus maze (EPM) test, and observed a modest, but significant, reduction in time spent in the covered arms of the elevated maze in SNI mice receiving M1 tDCS as compared to sham-treated mice, suggesting a mild reduction in anxiety in neuropathic mice (Fig. 4D).

### Analysis of impact of prefrontal tDCS to the responsivity of diverse brain regions to mechanical stimulation in the state of neuropathic mechanical allodynia

**Figure 5:**
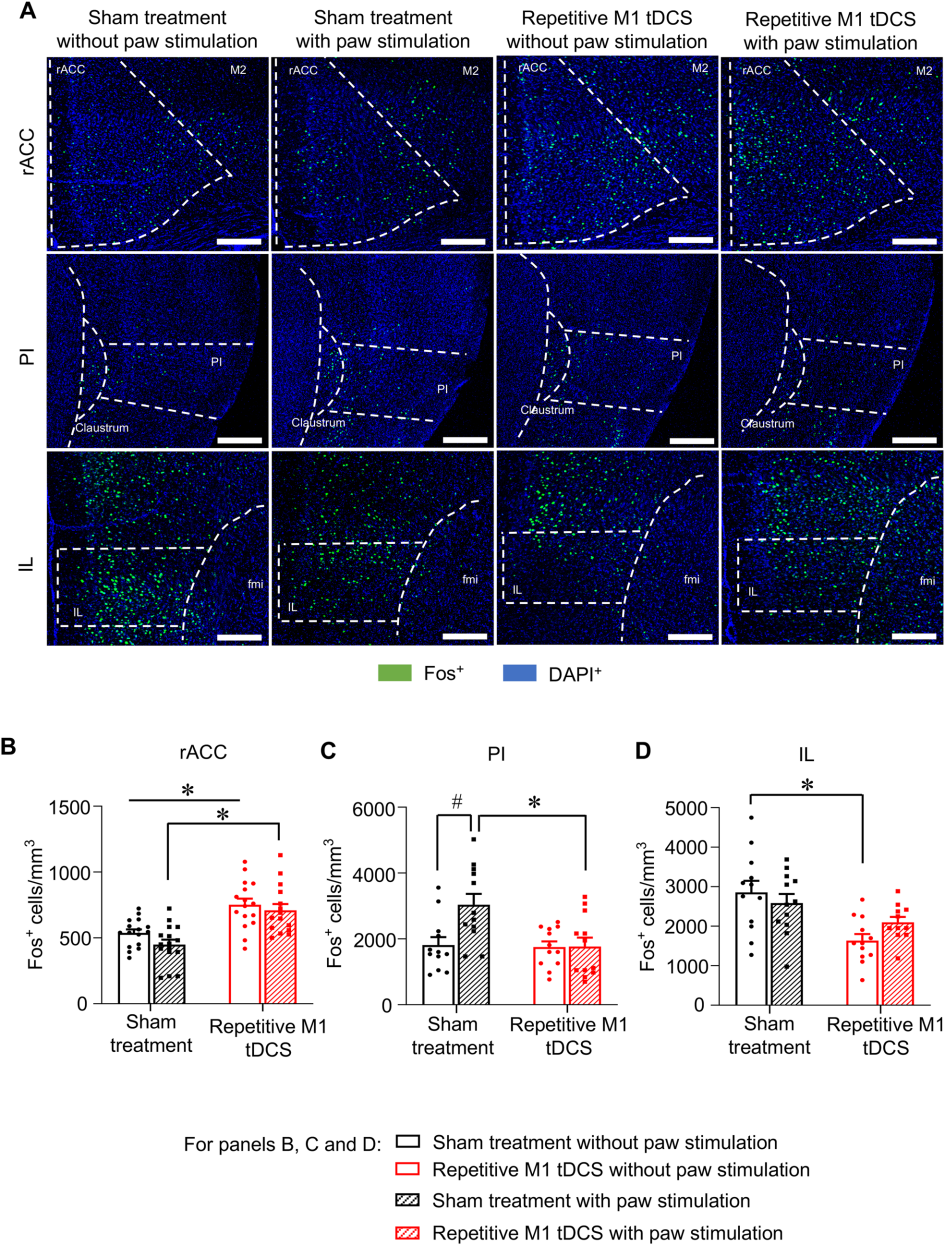
Comparison of brain regions activated by hind paw stimulation with low intensity mechanical force (0.16 g; corresponding to neuropathic mechanical allodynia) at chronic stages of neuropathic pain (43 days post-nerve injury) in SNI mice receiving repetitive M1 tDCS or 0 mA sham stimulation over 35–39 days post-nerve injury. The control group received no mechanical stimulation (control). (**A**) Typical examples of immunohistochemistry for the neuronal activity marker protein Fos with nuclear counterstaining with DAPI (scale bar = 100 µm), images were acquired with a confocal laser-scanning microscope (Leica LAS X, version 3.3.0), imported to Fiji-Image J software (version 1.50b). (**B**–**D**) Quantitative analysis of Fos-expressing cells over the rostral anterior cingulate cortex (rACC, **B**), posterior insula (PI, **C**) and infralimbic cortex (IL, **D**); n = 15–16 sections from 4 mice per group, ANOVA for random measures was performed, followed by Sidak’s test for multiple comparisons. *Indicates *p* < 0.05 as compared to the corresponding sham treatment group, # indicates *p* < 0.05 as compared to control in the sham treatment group and repetitive M1 tDCS group. Data are represented as mean ± S.E.M.

**Figure 6:**
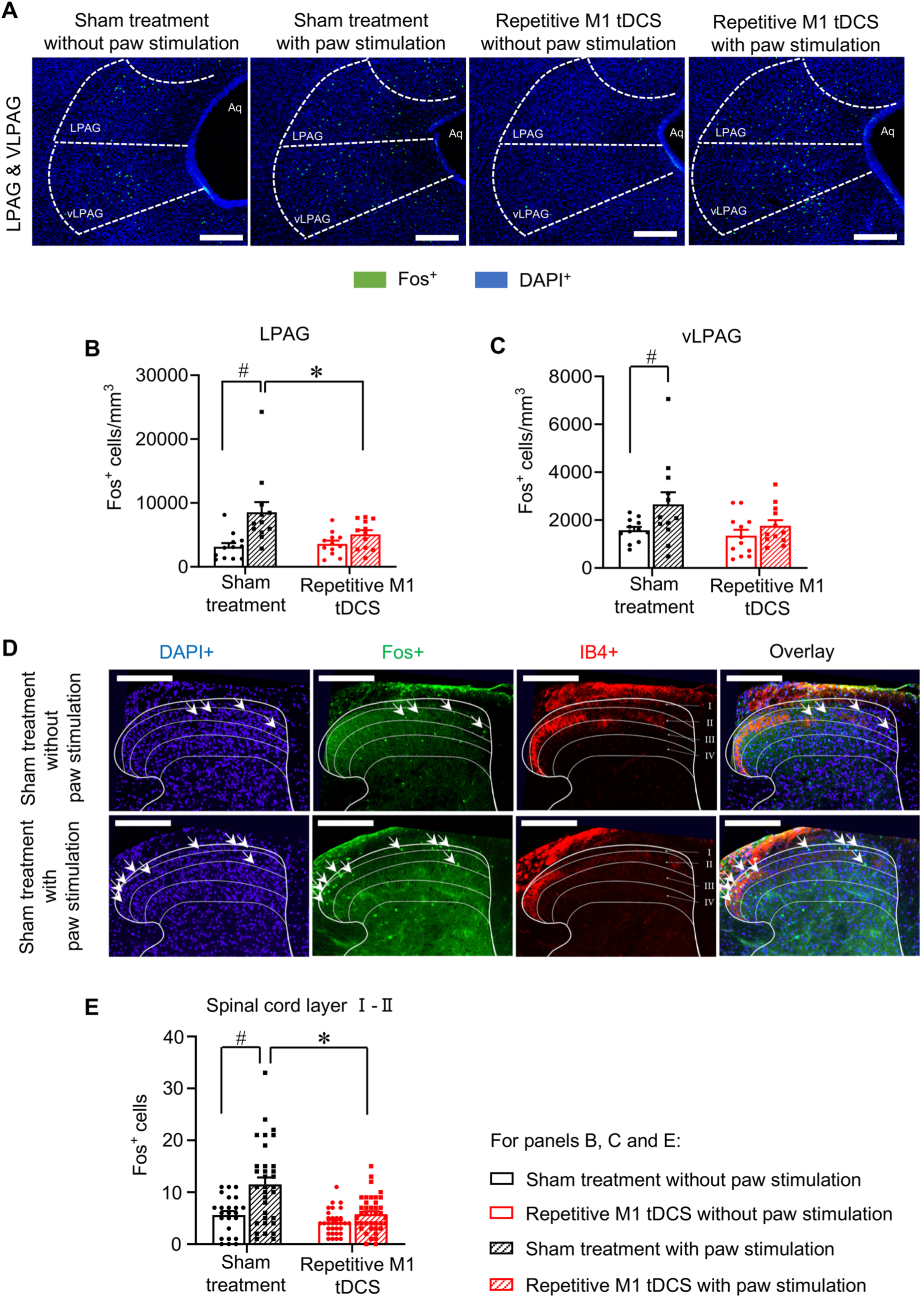
Comparison of key, pain-related brainstem nuclei and lumbar spinal cord laminae activated by hind paw stimulation with low intensity mechanical force (0.16 g) at chronic stages of neuropathic pain (43 days post-nerve injury) in SNI mice receiving repetitive M1 tDCS or 0 mA sham stimulation over 35–39 days post-nerve injury. The control group received no mechanical stimulation (control). (**A**) Typical examples of immunohistochemistry for the neuronal activity marker protein Fos with nuclear counterstaining with DAPI (scale bar = 100 µm) over ventrolateral periaqueductal grey (vlPAG) and lateral PAG (LPAG). (**B,C**) Quantitative analysis of Fos-expressing cells over LPAG (**B**) and vlPAG (**C**); n = 12 sections from 4 mice in the repetitive M1 tDCS group (with mechanical stimulation) and the sham treatment group (with or without mechanical stimulation), n = 9 sections from 3 mice in repetitive M1 tDCS group (without mechanical stimulation). (**D**,**E**) Typical examples of Fos immunohistochemistry with DAPI counterstaining (**D**; scale bar = 200 µm) or quantitative summary (**E**) over superficial laminae (I and II) in L3–L5 lumbar spinal cord segments; n = 6–12 sections were processed from each of 4 mice per group. Images were acquired with a confocal laser-scanning microscope (Leica LAS X, version 3.3.0), imported to Fiji-Image J software (version 1.50b). In all panels, ANOVA for random measures was performed, followed by Sidak’s test for multiple comparisons. *Indicates *p* < 0.05 as compared to the corresponding sham treatment group, # indicates *p* < 0.05 as compared to control in the sham treatment group and the repetitive M1 tDCS group, respectively. Data are represented as mean ± S.E.M.

Previous studies have reported changes in baseline (i.e. resting activity) of several brain regions upon M1 tDCS; however, little is known about how recruitment of brain centers to sensory stimuli is altered. Given the strong inhibition of mechanical allodynia observed in behavioral experiments, it is also important to address how responsivity of brain regions in the context of innocuous mechanical stimulation is altered over repetitive M1 tDCS at chronic stages of neuropathy. We used the expression of Fos protein, the product of induction of the immediate early gene *c-fos* in response to synaptic activity and neuronal activation, as a neurochemical marker of neuronal activation in a window of 45 min–1 h post-mechanical stimulation^32,33^. SNI mice were therefore divided into 4 groups, two to study baseline Fos expression (control, Fig. 5) and two to analyze Fos expression in response to hind paw stimulation with 0.16 g von Frey filament, with one group each receiving sham stimulation or repetitive M1 tDCS (Fig. 5). Analysis of Fos expression was performed at 4 days after the last cycle of stimulation (i.e. at a time point when protection against mechanical allodynia is strong in the tDCS group), which amounted to 43 days post-SNI (i.e. a chronic neuropathic pain stage). In both sham-treated and M1 tDCS mice, the innocuous stimulus of 0.16 g did not elicit any change over baseline Fos expression in the rostral anterior cingulate cortex (rACC; Fig. 5A,B), infralimbic cortex (IL; Fig. 5A,D), midcingulate cortex (MCC; Suppl. Figure 2A), M1 and M2 motor cortices (Suppl. Figure 2B,C), hindlimb area of the primary somatosensory cortex (S1HL; Suppl. Figure 2D), prelimbic cortex (PrL; Suppl. Figure 2E) and the ventroposterolateral and ventroposteromedial thalamic nuclei (VPL and VPM; Suppl. Figure 2F,G). In the brain, the posterior Insula (PI) (Fig. 5A,C), the lateral periaqueductal grey (LPAG) and the ventrolateral periaqueductal grey (vlPAG) (Fig. 6A–C) showed a clear increase in Fos expression upon innocuous mechanical paw stimulation of sham-treated SNI mice; this increase did not occur in SNI mice that received repetitive M1 tDCS (Figs. 5A,C, 6A–C). Thus, the only 3 regions (PI, LPAG, vlPAG) that showed a specific activation corresponding to mechanical allodynia in sham mice were not recruited in mice with M1 tDCS, highlighting their role in mechanisms underlying M1 tDCS-induced protection against neuropathic mechanical allodynia and aversion to light touch.

Given that previous studies on M1 tDCS have not employed repetitive blocks of tDCS and not addressed late, chronic stages of neuropathic pain, we also analyzed regions showing a change in resting activity (baseline Fos expression) in sham versus M1 tDCS groups at 43 days post-SNI. Surprisingly, only very few brain regions demonstrated a long-term change in resting activity after M1 tDCS. The rACC demonstrated increased baseline activation while the IL demonstrated decreased baseline activation in the M1 tDCS group as compared to sham-treated mice (Fig. 5B,D). All other regions tested demonstrated no changes in baseline Fos expression across the groups (Figs. 5, 6 and Suppl. Figure 2). This suggests a selective remodel of specific pathways, rather than a widespread alteration in resting activity of the brain, by repetitive tDCS in neuropathic mice.

Because the brainstem nuclei showing specific changes in evoked activity, namely the LPAG and vlPAG, play a role in descending modulation of spinal activity, we tested Fos-expressing in spinal nociceptive laminae (I and II; Fig. 6D,E) and deeper laminae that process nociceptive inputs (III and IV; Suppl. Figure 2H). Light touch specifically led increase in Fos-positive neurons in the superficial spinal laminae on the side ipsilateral to the nerve injury in sham-treated mice, which was completely abrogated in mice with M1 tDCS (Fig. 6D,E). In contrast, the deeper laminae showed no changes (Suppl. Figure 2H).

## Discussion

There is growing consensus on minimally-invasive brain stimulation as a viable and promising adjuvant or alternative to pharmacological treatments in chronic pain. However, there remains a need for optimizing protocols via mechanism-based approaches and rendering them applicable in routine clinical practice, and addressing these needs in human studies alone is difficult, cumbersome and costly^3,4^. A few previous studies in rats and mice have already established the utility and value of rodent models in understanding motor cortex stimulation-induced analgesia^15,16,18,34^. While a majority of these studies tested epidural stimulation and were largely restricted to single stimulation sessions and the analysis of nociceptive component of pain, this study advances knowledge by (i) testing the efficacy of transcranial anodal M1 stimulation, (ii) analyzing repetitive stimulation regimes recurring over different temporal phases of neuropathic pain, (iii) testing different sensory modalities as well as affect deviations in neuropathic pain and (iv) by delineating brain regions showing changes in baseline activity after M1 stimulation from those that are specifically recruited in mechanical allodynia.

Minimally-invasive brain stimulation offers flexibility in implementation of different cycles of stimulation. We therefore directly compared the impact of using multiple cycles over separate time frames within the same animal on behavioral parameters of neuropathic pain. Our data indicate that while a 5-day repetitive stimulation regime can afford significant protection against further progression and exacerbation of mechanical allodynia when applied over early stages of neuropathic pain, delivering additional cycles of stimulation at late stages of neuropathic pain to test offers stronger efficacy and enables reversing chronically established mechanical allodynia. Moreover, cold allodynia, a relentlessly unpleasant problem associated with diverse types of peripheral neuropathies^2^, was lowered by the recurrent stimulation regime, but not by a single 5-day stimulation regime. Importantly, introducing the second 5-day stimulation block was not associated with stronger side effects. Both regimes evoked the same short-lasting enhancement of locomotor abilities, consistent with analyses in the human context where measurements of motor evoked potential indicate that anodal tDCS alters the excitability of the M1 cortex, while cathodal tDCS causes inhibition^35^. In rodent experiments, there is always a concern that the use of withdrawal behavior as a parameter for studying sensory function is confounded by potential motor impairments caused by the test interventions^36^. Here, motor programs were facilitated, rather than inhibited, while withdrawal behavior to cold and mechanical stimuli decreased in tDCS-treated mice, indicating that these behavioral changes reflect antinociception rather than an interruption of motor function by M1 anodal tDCS. Moreover, the change in motor function was not evident from the day after stimulation onwards, while antinociception was observed for at least 9 days after the stimulation cycle. Importantly, we also tested voluntary, non-reflexive behaviors in this study, which give more confidence that the behavioral readouts represent a reflection of the subjective percept in the animals^36^. Importantly, we also report that the M1 tDCS regimen we employed is effective in completely abrogating pain-related aversion in neuropathic mice, which indicates relevance to the multidimensional experience of pain and to the human and clinical context. The observation that the M1 tDCS regimen was efficacious against both sensory qualities and aversive aspects of neuropathic pain and also partly alleviated anxiety is noteworthy, especially given the emerging view that brain circuits underlying these components are distinctive but may considerably overlap and interact in chronic pain states^37,38^.

A major hindrance in development of brain stimulation-based analgesic therapy has been given by gaps in knowledge about the brain sites and circuits underlying the mechanisms of action^3,4,10,11^. In studies involving epidural stimulation targeting the primary motor cortex via electrodes placed above the central sulcus, Garcia-Larrea & Peyron (2007)^10^ reported regional cerebral blood flow changes in the post-stimulation period (35 min–2 h), when analgesic effects were evident, located to the mid-cingulate and pregenual ACC, the dorsolateral prefrontal cortex, the orbitofrontal cortex, the thalamus and the PAG, suggesting alterations in the baseline (resting) activity of these regions following motor cortex stimulation^4,10^. Most of the insights on regions altered by electrical stimulation approaches in animal studies so far have come from studies on epidural or transdural motor cortex stimulation in neuropathic^15,18^ or healthy states^16^. A study employing bipolar electrodes implanted above the dura in rats using the chronic constriction injury model to deliver a single MCS session revealed increased expression of activity-related immediate early genes over the PAG, ACC and amygdala and decreased expression in the VPL/VPM after the single stimulation session^18^. However, much less is known about regional changes in brain activity following transcranial electrical stimulation over the M1 region and chronic, late stages of neuropathic pain have particularly not been well studied in animal models. We therefore studied the impact of M1 tDCS applied over very late stages post-SNI, which pertain closely to the clinical situation since patients typically only receive motor cortex stimulation when their neuropathic pain is chronically established and resistant to drug therapy^4,6,13^. Surprisingly, the regimen used for repetitive M1 tDCS in late stage neuropathic mice was only associated with an increase in Fos activation in the rostral ACC and a decrease in Fos activation in the infralimbic cortex; in contrast, a large number of pain-related areas tested, including thalamic nuclei, showed no changes over baseline. Taken together with the afore-mentioned analyses in rodent models, these results suggest that although a number of brain areas related to pain processing may develop changes in activity in the acute context of M1 stimulation, a major adaptation sets in over time, e.g. via activity-dependent homeostatic plasticity, and only particular circuits show sustained modulation after repeated stimulation over chronic periods of neuropathic pain. The finding that repetitive M1 tDCS induced a compelling change in the baseline activity of the infralimbic cortex is novel and interesting, especially since, in comparison with its prelimbic counterpart, the infralimbic cortex remains very poorly studied in the context of pain. Only two rodent studies have addressed its role in neuropathic pain, reporting negative results^39,40^, however, chronic stages of neuropathic pain are not addressed and very little is known about specific circuitry and neuronal populations. This is interesting in the context of our observation of a tonic decrease in infralimbic activity upon M1 tDCS, which may result in enhancement of the widely reported role of the prelimbic cortex activation in suppressing neuropathic pain^41^ via its projections to the PAG.

Upon considering the results of this study and previous literature together, an increase in resting activity parameters in the ACC emerges to be the most consistent change seen across acute and chronic stages of neuropathy in mouse models and also closely matches the observations from human studies on motor cortex stimulation^10,15,18^. Diverse subdivisions of the ACC have been implicated in both sensory and emotional aspects of pain^42,43^. The rACC, which was showed an enhancement in resting activity after M1 tDCS in mice in this study, corresponds most closely to the perigenual ACC division in human brain, which has been reported to show sustained activation in the period following motor cortex stimulation, both epidurally via anodal currents^10^ and via transcranial magnetic stimulation^44^. Given the facilitatory role ascribed to diverse ACC domains in pain and the observation of enhanced ACC activity in several chronic pain cohorts as well as animal models of pain^42,43^, the finding that ACC activity is enhanced upon repetitive M1 tDCS appears to be at odds with the analgesic phenotypes observed. However, there are also indications that implicate the ACC in inhibition of pain; for example, chronic back patients show a disrupted rACC connectivity with brain regions in the default mode brain network^45^. Moreover, while opioids can decrease blood flow changes and suppress pain-related potentials in the ACC, a paradoxical increase in these activity parameters in the ACC and the orbitofrontal cortex has also been reported in response to both opioid treatment and placebo analgesia^46^. It is likely that this heterogeniety is imparted by distinct functional subdivisions or ensembles of neurons within the ACC and their differential connectivity to target areas. For example, prefrontal cortical projections to reward pathway centres, such as the nucleus accumbens and the ventral tegmental area, have been implicated in pain affect but not in sensory modulation^47^, while prefrontal connections to the PAG are implicated in modulating nociception. These deserve further study in the context of M1 stimulation-evoked analgesia. Interestingly, in the time period following the end of a motor cortex stimulation session in human subjects, activity-related parameters are elevated in the PAG and there is a positive connectivity between the ACC and the PAG^10^. Moreover, electrophysiological studies in rats have indicated that the firing of PAG neurons is increased during the period of M1 stimulation^15^. While we did not observe an increase in resting activity in the PAG upon repetitive M1 tDCS at chronic stages of neuropathic pain, we noted that PAG responsitivity to mechanical stimuli is increased.

Importantly, this study did not restrict analysis to baseline (resting) activity, but provides key insights into regions underlying the reversal of mechanical allodynia in late stage neuropathic states. Activity markers, such as Fos, by virtue of their short life, enable dissecting out the patterns of activity that are relevant for a particular sensory response over backblock activity^48^. We were particularly interested in how responsivity to a low intensity mechanical stimulus, which is typically innocuous in baseline conditions but evokes unpleasant sensation and withdrawal in neuropathic states, is modified by mice receiving repetitive M1 tDCS. Strikingly, only the PI and two subdivisions of the PAG, namely vlPAG and the LPAG, were specifically recruited by this mechanical allodynia-associated stimulus in late stage neuropathic mice, which was fully abrogated by M1 tDCS. The PI is a highly critical cortical domain implicated in intensity encoding of pain^49^ and is one of the few brain regions which has been reported to evoke pain sensation upon acute stimulation in the human context^37^. The PI facilitates spinal nociception by projecting to serotonergic neurons in the raphe nuclei, which activate descending facilitatory pathways^50^. A recent study has demonstrated that serotonergic neurons in the raphe nuclei are activated by motor cortex stimulation in healthy, non-neuropathic rats^16^. Moreover, specific activation of a pathway from the PI to the central amygdaloid nuclei has been shown to modulate aversive and anxiety-like behaviours in the context of neuropathic pain^51^. Thus, the failure to recruit the PI by stimuli associated with mechanical allodynia in mice with M1 tDCS can contribute towards suppression of both, sensory and emotional components of neuropathic pain and anxiety.

Likewise, the LPAG and the vlPAG areas are considered to be the site of origin of descending inhibitory pathways to the spinal cord. Therefore, the observation that recruitment of the LPAG and the vlPAG subdivisions is blunted by M1 tDCS while mechanical allodynia is reversed may appear paradoxical at the first glance; moreover, this finding may also appear contrary to the overall increase in PAG noted in previous studies on M1 stimulation, although it should be noted that one study reported decrease in expression of an activity-related marker in the PAG upon M1 stimulation in healthy rats^34^. However, recent studies demonstrate that although global manipulations of PAG activity indeed suppress nociception, the PAG is not structurally and functionally homogenous with respect to pain and that there is a rich diversity of neurons differentially promoting or suppressing nociception within the vlPAG^52^. By virtue of modulating the activity of the serotonergic raphe nuclei and the adrenergic locus ceruleus, which are involved in both descending facilitation as well as descending inhibition, the role of the vlPAG is not only complex but also uniquely plastic. It has been suggested that in chronic pain states, there is a disbalance between descending inhibition and descending facilitation and a net shift towards facilitation of nociception^37^. Therefore, it will be imperative in future studies, when neurochemical markers for specific functional cellular subtypes within the microcircuit of the PAG become available, to address how specific neurons change their activity in response to M1 tDCS in chronic stages of neuropathic pain. Furthermore, the observation that repetitive M1 tDCS suppresses activation of ipsilateral spinal cord neurons in the superficial nociceptive laminae by light touch supports the notion that repetitive M1 tDCS inhibits descending facilitatory influences in chronic phases of neuropathic pain. This may be different from acute, single session stimulation, where M1 stimulation has been suggested to strengthen descending inhibition^18^.

In conclusion, the results of this study are predictive of enhanced therapeutic benefits upon implementing recurrent tDCS cycles, particularly from the point of view of reversing chronically established neuropathic pain. They show that repetitive M1 tDCS can significantly alleviate allodynia, aversion and anxiety by inducing compelling changes in the overall baseline activity of only a small number of brain regions and by strongly modulating the responsivity of key brain regions and spinal superficial laminae to mild sensory stimuli that evoke mechanical allodynia and aversion at chronic stages of neuropathic pain in mice.

## Materials and methods

### Animals

All experiments were conducted in C57BL/6 J mice (20–30 g) of both sexes at 8–20 weeks of age that were obtained from Janvier Labs. In total 70 animals were used and the gender ratio was balanced across all experiments. Mice of the same sex were housed together in groups of 2–3 per cage and kept under a 12 h light / dark cycle at controlled temperature (22 ± 2 °C), humidity (40–50%) with food and water provided ad libitum according to ARRIVE guidelines. All experimental procedures were approved by the local governing body (Regierungspräsidium Karlsruhe, Germany, Ref. 35-9185.81/G-205/14), and abided to German Law that regulates animal welfare and the protection of animals used for the scientific purpose (TierSchG, TierSchVersV).

### Experimental design

The animals were adapted to the holding environment for 2–3 days and left to recover for another 5 days following electrode holder implantation. The animals were randomly divided into two groups for mechanical and cold sensitivity testing (sham treatment, repetitive M1 tDCS), a further 4 groups for testing aversiveness to mechanical stimulation (sham lesion and sham treatment, sham lesion and repetitive tDCS, spared nerve lesion and sham treatment, spared nerve lesion and repetitive tDCS), and another four groups for the immunohistochemistry experiments (sham treatment without paw stimulation, sham treatment with paw stimulation, repetitive M1 tDCS without paw stimulation, repetitive M1 tDCS with paw stimulation). From 8 to 35 days following the spared nerve lesion animals underwent daily tDCS sessions for five consecutive days at an early and late stage of neuropathic pain, labelled Block I and Block II, respectively. Mechanical and cold sensitivity were assessed at 1 day before nerve lesioning, 1 day before the first tDCS treatment session in both blocks, and on defined days over a 16-day period following the final tDCS treatment session in each block. Motor function (open field test) and anxiety (elevated plus maze) were assessed separately in Blocks I and II, respectively. Aversiveness to mechanical stimulation with the place escape / avoidance paradigm test was assessed in a separate cohort of animals on 4 days following repetitive tDCS (16 days following the spared nerve lesion). For immunohistochemistry experiments, animals received mechanical von Frey stimulation on day 4 following the Block II repetitive tDCS sessions (43 days following the spared nerve lesion), were killed 1 h later by a high dose of carbon dioxide, and immediately perfused with formalin fixative. The experimenters were blinded to the identity of mice being analysed in behavioural and immunohistochemical analyses. Behavioural testing was performed during the light (day) phase.

### Electrode holder and surgical implantation

Electrode holders made of PEEK (polyetheretherketone) were designed to isolate a 2–3 mm^2^ narrow area of animal’s cranium, corresponding to the hindlimb of the primary motor cortex (M1) on the right hemisphere and the rostral tip of the prefrontal cortex (PFC) on the contralateral side (Fig. 1A). Animals were anesthetized with an intraperitoneal injection of Medetomidine (Alvetra, Neumünster, Germany; 0.3 mg/kg), Fentanyl (Janssen-Cilag, Neuss, Germany; 0.01 mg/kg) and Midazolam (Hameln Pharma Plus, Hameln, Germany; 4 mg/kg). The head was fixed in a stereotaxic alignment system (David Kopf Instruments, USA) and the scull exposed by standard surgical procedures. The anodal M1 electrode holder was centred at the anterior–posterior (AP) axis at − 0.25 mm relative to Bregma and the mediolateral (ML) axis at + 0.8 mm from the midline, with 15° clockwise rotation from the longitudinal AP axis. The cathodal electrode was centred at AP: + 3.9 mm, ML: − 0.95 mm. The electrode holders were cemented onto the skull with 3 layers of dental cement (Paladur, Heraeus Kulzer, Germany). A thin film of bone wax at the base of the electrode holder ensured isolated electrolyte contact via the central cavity with the scull bone. A mixture of Naloxone (Inresa Arzneimittel, Freiburg, Germany; 0.4 mg/kg), Flumazenil (Fresenius, Bad Homburg, Germany; 0.5 mg/kg), Antipamezol (Prodivet Pharmaceuticals, Belgium; 2.5 mg/kg), and Carprofen (Norbrook Laboratories, Northern Ireland; 5 mg/kg) were injected intraperitoneally to reverse the anaesthesia and provide postoperative pain relief. The animals were singly caged on a warm heating plate until full recovery. Coordinates for diverse brain regions in the study were based on the mouse brain atlas by Paxinos and Franklin^53^.

### Spared nerve injury (SNI)

Mice of both sexes were assigned randomly and equally to SNI and sham groups. At 7 days after implanting electrode holders, animals were anesthetized again with the Medetomidine / Midazolam / Fentanyl mixture (see above) and the sciatic nerve and its three branches (sural, common peroneal, and tibial) exposed via an incision of the lateral thigh skin. As described previously^30^, the common peroneal and tibial nerves were tightly ligated and cut distally, a 2–4 mm section was removed from the ligation with the sural nerve left intact. The sham operation proceeded similarly without any nerve damage.

### Transcranial current stimulation (tDCS)

All animals received 5 treatment sessions per block (15 min per day over 5 consecutive days), and were initiated at 8 days and at 35 days after the SNI surgery in Block I and Block II, respectively. The tDCS protocol was adapted from previous tDCS reports^54–56^. Mice were anesthetized with 3% isoflurane, the head fixed via the nosepiece of the stereotaxic mask (RWD Life Science Company, China), and a light sedation maintained with 1% isoflurane. Electrode holders were filled with saline and tungsten wire electrodes inserted with the M1 electrode serving as anode (+) and the rostral PFC electrode as cathode (−). Constant current at 0.35 mA was applied for 15 min via an A320 stimulus isolator (World Precision Instruments Inc., USA). Mice in the sham treatment group were subjected to the same procedures but without switching on the stimulator.

### Behavioural tests

Animals were acclimatized to the von Frey testing arena for 2 h the day before baseline and pre-tDCS test days as well as at 20–30 min before each testing session. Following a daily tDCS treatment course over 5 days, mechanical sensitivity and cold plate tests were performed 2 h after the fifth treatment session, and then on 2, 5, 9, and 16 days post treatment, respectively, in both Blocks. Animals were not acclimatized on the elevated plus maze, the open field arena, or the place escape/avoidance paradigm arena. Motor behaviour in the open field was tested in Block II at 2 h, 2 days, 5 days, 9 days and 16 days post treatment.

### von Frey filaments

Mechanical sensitivity testing was performed on an elevated grid (Ugo Basile Inc., Italy) by applying a set of von Frey filaments with increasing forces (0.04–2.00 g), to the affected hind paw using the up-down method described by Dixon^57^. Von Frey filaments were applied to the lateral plantar surface of the hind paw in a brief vertical upward motion. Withdrawal frequencies were determined from 5 applications per filament, with a minimal interval of 5 min between applications. Paw lifting was defined as a positive response.

### Cold plate test

Following mechanical sensitivity testing, Animals were placed on a circular cold metal surface (5 °C, Hot/Cold Plate 35100, Ugo Basile Inc., Italy) enclosed by a Perspex cylinder, and closely monitored in order to record the latency of the first nociceptive response (paw lifting, shaking, licking, or jumping). A 30 s cut-off was used to prevent potential injury to the paws. Animals were exposed only once per test day in a single trial to the cold plate.

### Open field test (OF)

Animals were placed at the centre of a square arena enclosed by black walls (40 × 40 cm, and 40 cm in height; Ugo Basile Inc., Italy) and were allowed to explore the arena freely for 10 min^48,58–61^ Animal movement was tracked via a video camera and ANY-maze software (Stoelting Co., Ireland), from which distance travelled and mean speed parameters were extracted.

### Elevated plus maze (EPM)

Anxiety-like behaviour was evaluated based on the cumulative exploration time spent by each mouse in the open zones of an EPM apparatus^62–65^. The maze consists of two open arms (5 cm wide and 35 cm long), and two closed arms of the same size with 15 cm high walls, symmetrically arranged around an open centre zone of 5 × 5 cm. The maze was elevated 50 cm above the floor and lighting was dimmed to a level that still allowed video tracking. The animals were placed in the centre of the maze and allowed to explore the environment freely for five minutes. The test was recorded with a USB camera and analysed using ANY-maze software to determine times spent in either the centre zone, and the open or closed arms.

### Place escape/avoidance paradigm (PEAP)

The aversiveness of evoked mechanical stimulation in neuropathic animals was assessed via PEAP two days after the last tDCS treatment session, which was modified from a previous study^66–68^. Animals were placed in an arena (22 cm width × 22 cm length × 12 cm height), which was divided into a closed chamber (the roof and walls were covered with black foil) and an open chamber covered with white foil connecting with a 3.5 cm opening in the dividing wall. Mice could choose freely between the chambers. The first 5 min out of 30 min served as an unstimulated reference baseline. Animals were then stimulated every 15 s for the remaining 25 min with a 0.07 g von Frey filament applied to the affected hind paw when the animal was in the closed (dark) chamber, while the contralateral hind paw was stimulated when the animal was in the open (bright) chamber. The entire period of testing was digitally recorded via a USB camera and analysed by ANY-maze software (Stoelting Co., Ireland).

### Immunohistochemistry

Repeated mechanical stimulation (0.16 g von Frey filament application, every 30 s over a 20 min period) of the injured hind paw was used to assess neuronal activity in response to a low intensity mechanical stimulation, which is normally innocuous, but associated with mechanical allodynia in nerve injured mice. Mechanical stimulation or no stimulation (control) was performed at 4 days after the last tDCS session on day 43 post-SNI. Animals were killed at 60 min after mechanical stimulation and perfused transcardially with phosphate-buffer saline (PBS; pH 7.4) followed by 10% formalin fixative solution (Merck, Germany). Brains were sectioned coronally at 50 µm were stained as previous described^48^ with primary anti-Fos (Rabbit-anti-Fos, 1:1,000, Abcam, ab190289, UK) and secondary antibody (Donkey anti-rabbit Alexa 488, 1:700, Invitrogen, USA). Frozen sections at 25 µm thickness from spinal segments (L3–L5) were processed similarly with the exception that antigen retrieval was performed via incubation in 1 mM EDTA solution prior to the actual staining procedure. Unspecific antigen blocking was achieved by incubation for 30 min in PBS-T containing 5%(v/v) donkey serum (Abcam, ab7475, UK). Rabbit-anti-Fos (1:1,000, Abcam, ab190289, UK) and biotinylated Isolectin B4 (IB4, 1:150, Sigma, USA) were applied for 1 h at room temperature followed by 48 h at 4 °C. Secondary donkey anti-rabbit Alexa 488-conjugated antibody (1:700, Invitrogen, USA) and Alexa 647-conjugated Streptavidin (1:200, Invitrogen, USA) diluted in blocking solution were employed, and nuclei were counterstained using Hoechst 33,342 (1:10,000, Molecular Probes, USA).

### Image acquisition and quantification

Labelled sections were imaged with a confocal laser-scanning microscope (10 X objective; Leica TCS SP8 AOBS, Germany) using identical illumination exposure parameters for all animals. Sequential line scans were used at a pixel resolution of 1024 × 1024 for brain sections and 4096 × 4096 for spinal cord sections. A montage of confocal image stacks was acquired over a depth of 20 µm. The maximum z-projection brain images were applied to automatically analyse by Fiji-Image J software (version 1.50b, National Institutes of Health, USA) with a thresholding approach (threshold > 30, pixel^2 size > 6, circularity 0.23–1.0) on 8-bit format images and data from the stereotaxic atlas^53^ was used to define region of interest outlines anatomically according to corresponding reference sections. After automatic counting, the results were further screened manually to exclude false positives. For spinal cord sections, the IB4-positive band was used as a demarcation for lamina II in the spinal dorsal horn^69^. Other laminae were manually annotated and Fos-positive cells were counted, as previously described^70^.

### Statistical analyses

A normal distribution of the data was verified in Prism (version 8.0, GraphPad Software Inc., USA) using the D’Agostino-Pearson omnibus K2 normality test and all data is expressed as mean ± standard error of the mean (S.E.M). Statistical significance of difference was determined using one-way ANOVA test, two-way repeated measures ANOVA with post hoc Sidak’s test, or Tukey’s tests enabling multiple comparisons using Prism. A *p* value of < 0.05 was considered significant in all tests.

## Supplementary Information

Supplementary Figures.

## Data Availability

The authors agree to make all primary data available upon request and will upload the data on a public repository post-publication.
