# Supplementary material for: Suppression of neuropathic pain and comorbidities by recurrent cycles of repetitive transcranial direct current motor cortex stimulation in mice
Source: Sci Rep. 2021 May 6;11:9735. doi: 10.1038/s41598-021-89122-6 (PMC8102487; doi:10.1038/s41598-021-89122-6)

Suppression of neuropathic pain and comorbidities by recurrent cycles of repetitive transcranial direct current motor cortex stimulation in mice

Zheng Gan[1, #], Han Li[1, #], Paul Vincent Naser[1], Manfred Josef Oswald[1] and Rohini Kuner[1,*]

[1]Institute of Pharmacology, Medical Faculty Heidelberg, Heidelberg University, Im Neuenheimer Feld 366, 69120 Heidelberg, Germany

[#] These authors contributed equally to this work.

*Correspondence should be addressed to R.K. (rohini.kuner@pharma.uni-heidelberg.de)

Supplementary figure legends:

Supplementary Fig. 1: (**A**) Electrode holder dimensions for the cranial placement of the anode and the cathode on mouse skull. (**B**) Analysis of plantar sensitivity to mechanical von Frey stimulation applying graded force in M1 tDCS or sham-treated mice prior to nerve injury. (**C**, **D**) Analysis of von Frey sensitivity in the same groups of mice at 6 days (**C**) or 34 days (**D**) post-nerve injury (SNI). but prior to repetitive M1 tDCS or 0 mA sham stimulation. In panels **B** and **C**, n = 7 mice/group; in panel **D**, n = 7 mice for the sham treatment group and n = 6 mice for repetitive M1 tDCS group; ANOVA for repeated measures was performed, followed by Sidak's test for multiple comparisons; n.s. represents non-significant differences between groups. Data are expressed as Mean ± S.E.M.

Supplementary Fig. 2: Quantitative analysis of Fos-expressing cells over diverse brain regions and the lumbar spinal cord upon hind paw stimulation with low intensity mechanical force (0.16 g; corresponding to neuropathic mechanical allodynia) at chronic stages of neuropathic pain (43 days post-nerve injury) in SNI mice that received either repetitive M1 tDCS or 0 mA sham stimulation over 35-39 days post-nerve injury. Shown are analyses over the mid-cingulate cortex (MCC, **A**), primary motor cortex (M1, **B**), secondary motor cortex (M2, **C**), primary somatosensory cortex, hindlimb region (S1HL, **D**),

prelimbic cortex (PrL, **E**), ventral posterolateral thalamic nucleus (VPL, **F**), ventral posteromedial thalamic nucleus (VPM, **G**) and the deeper spinal laminae III and IV (**H**). In panel **A** to **G**, n = 9 sections from 3 mice in repetitive M1 tDCS group (without mechanical stimulation) and n = 11-12 sections from 4 mice in each of the other groups. In panel **H**, n = 6-10 sections from 4 mice per group; ANOVA for random measures was performed, followed by Sidak's test for multiple comparisons, n.s. represents non-significant differences between groups. Data are expressed as Mean ± S.E.M.

Supplementary Fig.1

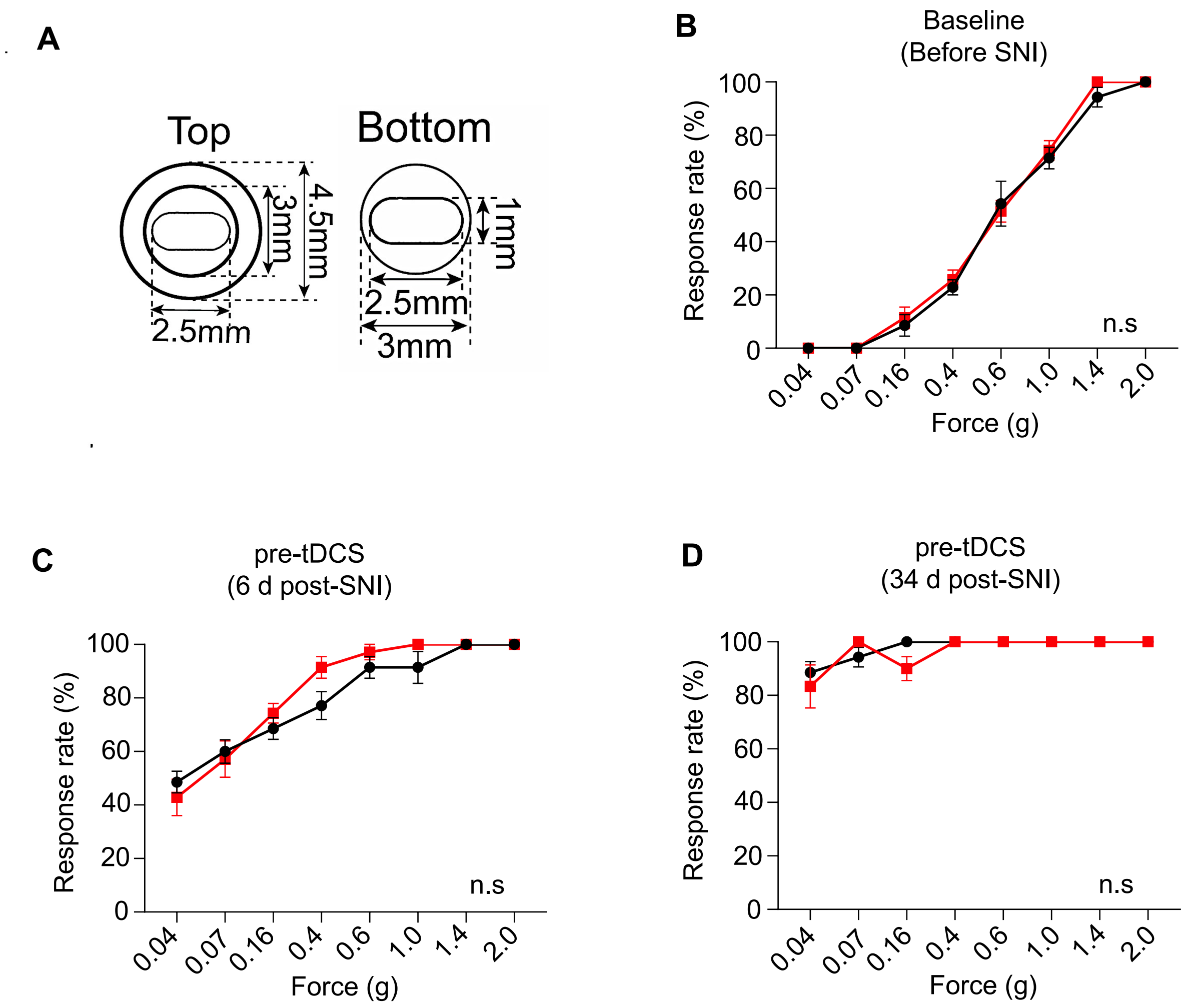

For panels B, C and D: ● Sham treatment ■ Repetitive M1 tDCS

Supplementary Fig.2

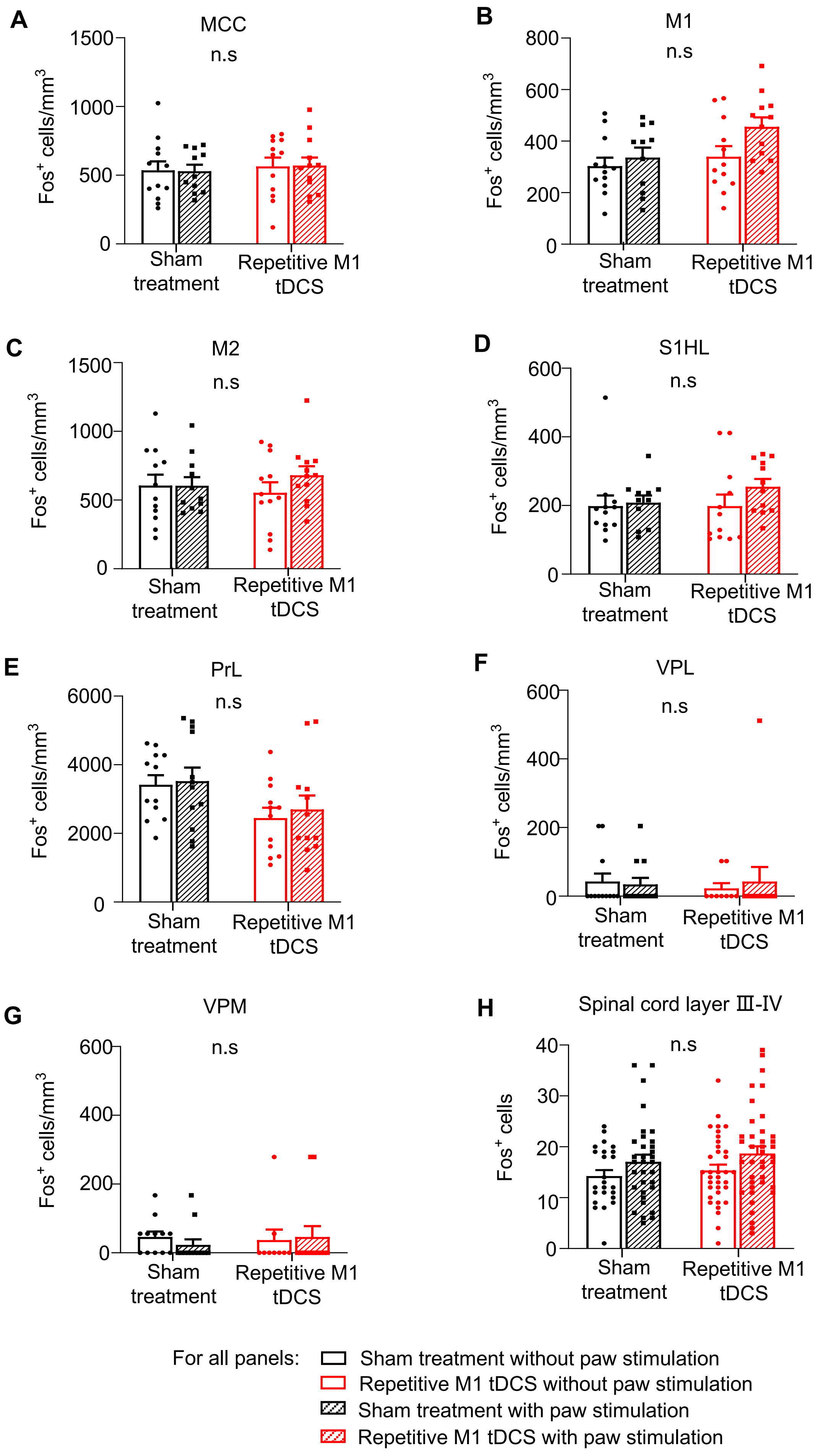

Supplement: Supplementary file 1 — Supplementary Figures. [file 41598_2021_89122_MOESM1_ESM.pdf]
